# Motivating factors and barriers towards exercise in severe mental illness: a systematic review and meta-analysis

**DOI:** 10.1017/S0033291716001732

**Published:** 2016-08-09

**Authors:** J. Firth, S. Rosenbaum, B. Stubbs, P. Gorczynski, A. R. Yung, D. Vancampfort

**Affiliations:** 1 Institute of Brain, Behaviour and Mental Health, University of Manchester, UK; 2 Department of Exercise Physiology, School of Medical Sciences, Faculty of Medicine, University of New South Wales, Australia; 3 Physiotherapy Department, South London and Maudsley NHS Foundation Trust, UK; 4 Health Service and Population Research Department, Institute of Psychiatry, Psychology and Neuroscience, King's College London, UK; 5 Department of Sport and Exercise Science, University of Portsmouth, UK; 6 Orygen Youth Health Research Centre, University of Melbourne, Australia; 7 KU Leuven Department of Rehabilitation Sciences, Leuven, Belgium; 8 KU Leuven Department of Neurosciences, UPC KU Leuven, Belgium

**Keywords:** Exercise, physical activity, physical health, psychosis, schizophrenia

## Abstract

Exercise can improve clinical outcomes in people with severe mental illness (SMI). However, this population typically engages in low levels of physical activity with poor adherence to exercise interventions. Understanding the motivating factors and barriers towards exercise for people with SMI would help to maximize exercise participation. A search of major electronic databases was conducted from inception until May 2016. Quantitative studies providing proportional data on the motivating factors and/or barriers towards exercise among patients with SMI were eligible. Random-effects meta-analyses were undertaken to calculate proportional data and 95% confidence intervals (CI) for motivating factors and barriers toward exercise. From 1468 studies, 12 independent studies of 6431 psychiatric patients were eligible for inclusion. Meta-analyses showed that 91% of people with SMI endorsed ‘improving health’ as a reason for exercise (*N* = 6, *n* = 790, 95% CI 80–94). Among specific aspects of health and well-being, the most common motivations were ‘losing weight’ (83% of patients), ‘improving mood’ (81%) and ‘reducing stress’ (78%). However, low mood and stress were also identified as the most prevalent barriers towards exercise (61% of patients), followed by ‘lack of support’ (50%). Many of the desirable outcomes of exercise for people with SMI, such as mood improvement, stress reduction and increased energy, are inversely related to the barriers of depression, stress and fatigue which frequently restrict their participation in exercise. Providing patients with professional support to identify and achieve their exercise goals may enable them to overcome psychological barriers, and maintain motivation towards regular physical activity.

## Introduction

People with severe mental illness (SMI) experience a premature mortality of around 15–20 years, largely due to inequalities in physical health (Ribe *et al.* 2014). For instance, people with SMI have a significantly higher risk of obesity, hyperglycaemia and metabolic syndrome, all of which contribute towards the development of cardiovascular diseases (Gardner-Sood *et al.* 2015). Many of these physical health issues are related to modifiable risk factors which can be treated and attenuated through lifestyle changes, including exercise and diet (McNamee *et al.* 2013; Curtis *et al.* 2016). This is particularly important for those receiving antipsychotic treatment since these medications greatly increase cardio-metabolic risk when combined with a sedentary lifestyle (McNamee *et al.* 2013; Vancampfort *et al.* 2015*b*).

People with SMI engage in significantly less vigorous exercise, and significantly greater amounts of sedentary behaviour than health controls (Stubbs *et al.* 2016*a*, *b*; Vancampfort *et al.* 2016*a*). This inactivity is predictive of a range of adverse health outcomes including obesity, diabetes and medical co-morbidity among people with SMI (Vancampfort *et al.* 2013*a*, *b*; Suetani *et al.* 2016). It is also associated with more severe negative symptoms and poor socio-occupational functioning (Vancampfort *et al.* 2012; Suetani *et al.* 2016).

An increasing body of research demonstrates that exercise interventions can improve physical health and reduce psychiatric symptoms in people with major depression and psychotic disorders (Rosenbaum *et al.* 2014; Firth *et al.* 2015). Exercise has also been found to reduce negative symptoms and cognitive deficits in schizophrenia (Firth *et al.* 2015; Kimhy *et al.* 2015); aspects of the illness which are often left untreated and particularly influential on long-term functioning (Galletly, 2009; Arango *et al.* 2013). Thus, proper implementation of exercise within the care of people with SMI could reduce cardio-metabolic risk and the associated mortality, while also facilitating functional recovery.

The optimal modality of exercise interventions for people with SMI is yet to be established. A recent meta-analysis suggests that various exercise modalities can be effective for improving outcomes in SMI, although only if a sufficient total volume of activity is achieved (Firth *et al.* 2015). Clinical trials have also found that significant benefits for depressive and psychotic symptoms only occur among participants who achieve sufficient amounts of exercise (Hoffman *et al.* 2011; Scheewe *et al.* 2013). Therefore, training programmes which can maximize adherence to exercise in SMI may be the most effective.

Meta-syntheses of the qualitative literature have previously examined the factors which may encourage or prevent exercise participation among people with SMI (Mason & Holt, 2012; Soundy *et al.* 2014*a*). For instance, improving self-identity and body image is a valued outcome of exercise programmes, while the sedative effects of psychotropic medications can inhibit physical activity (Mason & Holt, 2012; Soundy *et al.* 2014*a*). Although valuable, qualitative investigations can be influenced by interviewers’ biases, and results may only represent a subset of the population. Data from survey-based studies may therefore provide a more accurate representation of the entire patient group.

Improving our understanding of desired outcomes of exercise among people with SMI could enhance health promotion initiatives, and inform the development of interventions that are both motivating and rewarding for patients. Furthermore, determining the most common barriers would help to optimize resource allocation when delivering exercise services in clinical practice. Thus, we conducted a systematic review of studies reporting quantitative data on motivating factors and barriers towards exercise for people with SMI. We also quantified patients’ responses in these surveys using meta-analytical techniques to determine which were most pertinent for this patient group.

## Method

### Search strategy and selection criteria

An electronic database search of Ovid Medline, Allied and Complementary Medicine Database (AMED), PsycINFO, EMBASE, and the Health Management Information Consortium (HMIC) database, using the search algorithm: ‘exercise’ or ‘physical activity’ or ‘sport*’ AND ‘psychiatric’ or ‘severe mental’ or ‘serious mental’ or ‘schizophrenia’ or ‘psychosis’ or ‘bipolar’ or ‘manic depress*’ or ‘major depress*’ or ‘clinical depress*’ or ‘depressive disorder’ AND ‘motiv*’ or ‘barriers’ or ‘incentives’ or ‘attitudes’ or ‘preferences’ or ‘advantages’ or ‘disadvantages’ was conducted in May 2016, considering articles published from database inception. A search of Google Scholar was conducted using the same key words to identify any additional relevant articles. The reference lists of retrieved articles were also searched.

Only English-language research articles in peer-reviewed journals were included in this review. Eligible samples were those in which >80% of the sample had a diagnosis of a SMI (i.e. schizophrenia, schizoaffective disorder, other psychotic disorders, bipolar disorder or major depressive disorder) and/or were currently receiving treatment for SMI. Studies which inferred the presence of SMI solely from participants’ response to screening questionnaires were excluded if no diagnosis or current treatment for SMI could be confirmed. Eligible studies were those reporting proportional data on motivating factors and/or barriers towards physical activity among people with SMI, from questionnaires, surveys or other quantitative methods. Studies which used only qualitative methods were not eligible for inclusion, as these have been comprehensively reviewed elsewhere (Mason & Holt 2012; Soundy *et al.* 2014*a*). ‘Motivating factors’ were defined as any outcome of exercise perceived by patients to be a reason for increasing physical activity. ‘Barriers’ were defined as any physiological, psychological or socio-ecological conditions reported to reduce patients’ participation in exercise.

### Data extraction and data analysis

Articles were screened by two reviewers (J.F. and S.R.) to assess eligibility. Disagreements on eligibility were resolved through discussion. A systematic tool was developed (see Supplementary Table S1) to extract all relevant quantitative data from each study into the following categories:

(1) Motivating factors for exercise
  (*a*) *Physical:* physical health; fitness; strength; weight loss.
  (*b*) *Psychological:* well-being; enjoyment; reduce distress; mood; self-esteem.
  (*c*) *Socio-ecological:* socializing; health professional advice; routine.
(2) Barriers to exercise
  (*a*) *Physical: physical* illness; tiredness/fatigue.
  (*b*) *Psychological: distress*; depression; motivational; self-confidence; safety.
  (*c*) *Socio-ecological:* cost; access to facilities; time; support; insufficient information.

Information on study characteristics (sample size, demographics, location, care setting) was also extracted from each study, and is summarized in Table 1.

**Table 1.** Responses to survey items on motivating factors for exercise among people with severe mental illness

Category	Survey item	Average response^a^
(1) Physical health factors		
*General health*		
Bassilios *et al.* (2014)	Exercise for physical benefits	84% of those intending to exercise
Carpiniello *et al.* (2013)	Exercise is important for physical health	**88% agreed**
Deighton & Addington (2014)	Exercise will make me healthier	**95% agreed**
Faulkner *et al.* (2007)	Improve my health or reduce my risk of disease	Rated 4.3/5 for importance
Fraser *et al*. (2015)	To maintain good health	**98% agreed**
Gorczynski *et al.* (2010)	It would improve my health	Rated 4.3/5 on importance scale
Sylvia *et al.* (2009)	Exercise is beneficial for my physical health	**100% agreed**
Ussher (2007)	Exercise is important for physical health	**90% agreed**
Wynaden *et al.* (2012)	‘Why do you attend the gym?’	**61% said ‘to stay healthy’**
*Fitness/energy*		
Deighton & Addington (2014)	I will have more energy	**75% agreed**
Faulkner *et al.* (2007)	It would increase my energy levels	Rated 4.2/5 for importance
Firth *et al.* (2016*a*–*c*)	To increase fitness/energy	**68% agreed**
Fraser *et al*. (2015)	To improve my energy levels	**88% agreed**
Gorczynski *et al.* (2010)	It would help me to stay fit	Rated 4.2/5 on importance scale
Gorczynski *et al.* (2010)	I would have more energy	Rated 3.9/5 on importance scale
Kane *et al.* (2012)	Fitness	Rated 6/7 as a motivating factor
Sylvia *et al.* (2009)	Exercise improves my cardiovascular fitness	**78% agreed**. Avg. rating = 7.7/10
Wynaden *et al.* (2012)	‘Why do you attend the gym?’ (open-answer)	**59% said ‘to get fit’**
*Strength*		
Deighton & Addington (2014)	Exercise makes me feel strong	**83% agreed**
Firth *et al.* (2016*a*–*c*)	To increase sporting ability/strength	**50% agreed**
Fraser *et al*. (2015)	To build up my strength	**81% agreed**
*Body weight*		
Deighton & Addington (2014)	Exercise will help me lose weight	**80% agreed**
Faulkner *et al.* (2007)	It would help control my weight	Rated 3.8/5 for importance
Firth *et al.* (2016*a*–*c*)	To lose weight	**61% agreed**
Fraser *et al*. (2015)	To control my weight	**98% agreed**
*Appearance*		
Deighton & Addington (2014)	I will look better	**85% agreed**
Faulkner *et al.* (2007)	It would improve my muscle tone	Rated 4.2/5 for importance
Firth *et al.* (2016*a*–*c*)	To increase muscle tone	50% agreed
Fraser *et al*. (2015)	To improve my appearance	**64% agreed**
Kane *et al.* (2012)	Appearance	Rated 5.5/7 as a motivating factor
Sylvia *et al.* (2009)	Exercise improves my body shape and/or tone	**82% agreed.** Avg. rating = 8.3/10
(2) Psychological factors		
*General well-being*		
Bassilios *et al.* (2014)	Exercise for psychological benefits	27% of those intending to exercise
Carpiniello *et al.* (2013)	Exercise is important for mental health	**85% agreed**
Deighton & Addington (2014)	Exercising makes me feel better	**90% agreed**
Fraser *et al*. (2015)	Beneficial for managing psychological well-being	**95% agreed**
Fraser *et al*. (2015)	To give me space to think	73% agreed
Sylvia *et al.* (2009)	Exercise is beneficial to my mental health	**99% agreed**
Ussher (2007)	Exercise is important for mental health	**72% agreed**
Wynaden *et al.* (2012)	‘Why do you attend the gym?’	**38% ‘to help psychiatric problems’**
*Enjoyment*		
Carpiniello *et al.* (2013)	Enjoys exercise very much so or extremely so	**30% agreed**
Deighton & Addington (2014)	I will have fun	**85% agreed**
Firth *et al.* (2016*a*–*c*)	For having fun	**54% agreed**
Fraser *et al*. (2015)	Because I enjoy exercising	**54% agreed**
Gorczynski *et al.* (2010)	I would have fun	Rated 4/5 on importance scale
Kane *et al.* (2012)	Interest in exercise	Rated 5/7 as a motivating factor
Sylvia *et al.* (2009)	I have fun exercising	**46% agreed.** Avg. rating = 5.6/10
Ussher (2007)	Enjoys exercise very much so or extremely so	**57% agreed**
Wynaden *et al.* (2012)	‘Why do you attend the gym?’	**57% for ‘enjoyment’**
*Emotions and mood*		
Deighton & Addington (2014)	Exercise helps me manage my mood	**75% agreed**
Fraser *et al*. (2015)	To improve my emotional well-being	**94% agreed**
Sylvia *et al.* (2009)	It improves my mood and ability to cope with stress	**70% agreed.** Avg. rating = 7.1/10
*Reducing stress*		
Deighton & Addington (2014)	Exercise helps me manage stress	**85% agreed**
Faulkner *et al.* (2007)	It would help me feel less tense or stressed	Rated 4.1/5 for importance
Faulkner *et al.* (2007)	It would help me feel less angry or irritable	Rated 3.9/5 for importance
Faulkner *et al.* (2007)	It would take my mind off things	Rated 4/5 for importance
Firth *et al.* (2016*a*–*c*)	Taking your mind off things	64% agreed
Fraser *et al*. (2015)	To help manage my stress	**95% agreed**
Sylvia *et al.* (2009)	It improves my mood and ability to cope with stress	**70% agreed.** Avg. rating = 7.1/10
Wynaden *et al.* (2012)	‘Why do you attend the gym?’	**54% ‘to reduce stress’**
*Self-confidence*		
Deighton & Addington (2014)	Exercise makes me feel more self-confident	88% agreed
Deighton & Addington (2014)	I will feel better about myself	90% agreed
Faulkner *et al.* (2007)	It would improve how I feel about myself	Rated 4.4/5 for importance
Firth *et al.* (2015)	Being more confident in a gym	64% agreed
Gorczynski *et al.* (2010)	I would feel better about myself	Rated 3.9/5 on importance scale
Sylvia *et al.* (2009)	Exercise makes me feel good about myself	Rated 7.54/10 for relevance
*Sleep*		
Deighton & Addington (2014)	I will sleep better	**80% agreed**
Faulkner *et al.* (2007)	It would help me sleep better	Rated 3.9/5 for importance
Fraser *et al*. (2015)	It helps me sleep better	**79% agreed**
Sylvia *et al.* (2009)	I can sleep better if I exercise	**59% agreed.** Avg. rating = 6.6/10
(3) Socio-ecological factors		
*Social aspects*		
Firth *et al.* (2016*a*–*c*)	Meeting new people’	**29% agreed**
Fraser *et al*. (2015)	I enjoy the social aspects	**27% agreed**
Gorczynski *et al.* (2010)	People important to me would be happy if I did	Rated 3.7/5 on importance scale
Kane *et al.* (2012)	Social	Rated 3.4/7 as a motivating factor
Sylvia *et al.* (2009)	Exercising is a chance for me to see people	**27% agreed.** Avg. rating = 4.1/10
*Professional support*		
Carpiniello *et al.* (2013)	Would exercise more with doctors’ advice	63% agreed
Carpiniello *et al.* (2013)	Instructor's help would increase levels of exercise	68% agreed
Fraser *et al*. (2015)	I exercise because my doctor advised me to	61% agreed
Ussher (2007)	Would exercise more with doctors’ advice	58% agreed
Ussher (2007)	Instructor's help would increase levels of exercise	58% agreed
Daily routine		
Sylvia *et al.* (2009)	I have nothing better to do with my time	Rated 2.87/10 for relevance
Sylvia *et al.* (2009)	Exercise helps to structure my day	Rated 4.96/10 for relevance
Wynaden *et al.* (2012)	‘Why do you attend the gym?’	45% ‘to get into a routine’
Wynaden *et al.* (2012)	‘Why do you attend the gym?’	42% ‘to pass time’

a **Bold** indicates inclusion in meta-analyses.

### Data synthesis and meta-analysis

We sought to establish the overall prevalence of motivating factors or barriers towards exercise proportion among people with SMI. Therefore, where any specific motivating factor/barrier had been examined by ⩾3 independent studies, data was pooled using proportional meta-analysis in StatsDirect 2.7 (StatsDirect, 2005). A random-effects model was applied in all meta-analyses, in order to account for expected heterogeneity between studies (DerSimonian & Laird, 1986). The degree of variance between studies was assessed with Cochran's *Q* and indexed as *I*^2^, which estimates the amount of variance caused by between-study heterogeneity, rather than chance. As wording of questions can differ between studies, combinability of study data for meta-analyses was first established through agreed selection by two reviewers (J.F. and S.R.).

### Search results

**Fig. 1.**
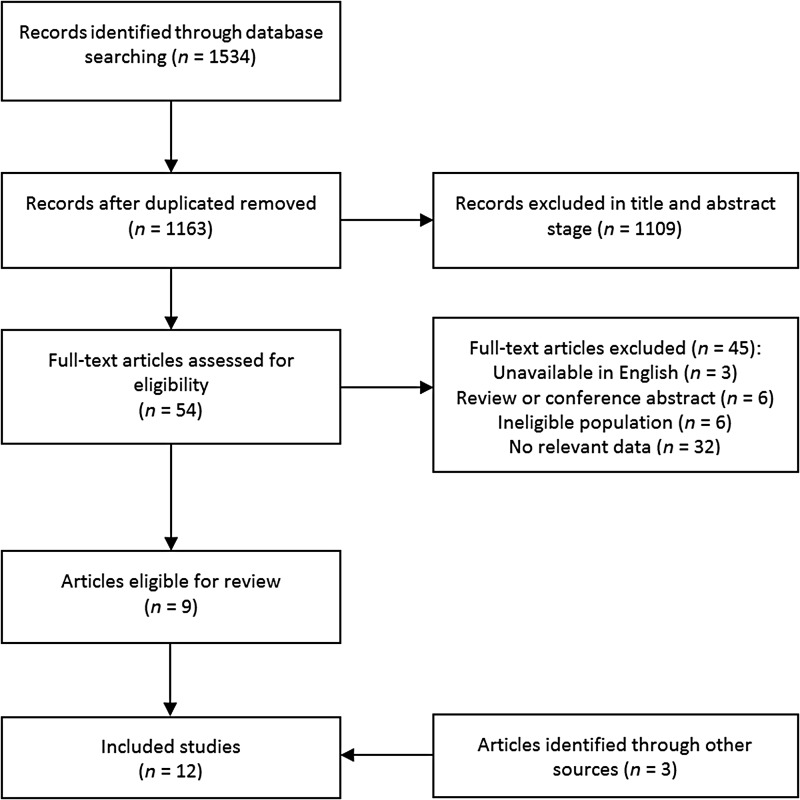
PRISMA flow diagram of systematic search and study selection.

Fig. 1 shows the full study selection process. The initial database search returned 1534 results. This was reduced to 1163 after duplicates were removed. A further 1109 articles were excluded after reviewing the titles and abstracts for eligibility. Full text versions were retrieved for 54 articles, of which nine were eligible for inclusion. A further three articles were identified from a similar search of Google Scholar. A total of 12 different studies articles, each with unique samples were eligible for inclusion (Faulkner *et al.* 2007; Ussher, 2007; Sylvia *et al.* 2009; Gorczynski *et al.* 2010; Kane *et al.* 2012; Wynaden *et al.* 2012; Carpiniello *et al.* 2013; Bassilios *et al.* 2014; Deighton & Addington 2014; Fraser *et al.* 2015; Klingaman *et al.* 2014; Firth *et al.* 2016*a*). Additional data was obtained for four studies from the corresponding authors (Sylvia *et al.* 2009; Gorczynski *et al.* 2010; Deighton & Addington, 2014; Firth *et al.* 2016*a*).

### Included studies and participant details

Characteristics of included studies are detailed in Supplementary Table S2. Three were conducted in the United States, three in Canada, three in Australia, two in the UK, and one in Italy. There were a total of 6431 psychiatric patients within these studies; 85.5% with schizophrenia, 6.2% with an unspecified SMI, 2.3% with bipolar or major depression, and 6% other/unknown diagnosis. Where specified, 65% were community-based outpatients while 35% were inpatients within psychiatric units. The median age was 42.6 years (range = 19.8–55 years). Samples ranged from 26–86% male (median = 62%). Of 5757 subjects, 50% belonged to minority groups within their respective countries, while 50% were white. Five studies (*n* = 470) also reported employment, showing that 68% of participants were unemployed. All survey items which were combined for meta-analyses are highlighted in Tables 1 and 2.

**Table 2.** Responses to items on barriers towards exercise among people with SMI

Category	Survey item	Average response^a^
(1) Physical barriers		
*Poor physical health*		
Bassilios *et al.* (2014)	Physical health problems as a barrier	28% of non-vigorous exercisers
Carpiniello *et al.* (2013)	Illness	**19.6% agreed**
Fraser *et al*. (2015)	Physical health problems	**44% agreed**
Sylvia *et al.* (2009)	Exercise will not change my physical health	Avg. rating = 1.7/10
Ussher (2007)	Illness	**15% agreed**
Fraser *et al*. (2015)	Feel too unwell	60% agreed
*Tiredness/low energy*		
Carpiniello *et al.* (2013)	Too tired	**38.4% agreed**
Deighton & Addington (2014)	Lack of energy	‘Sometimes a barrier’
Faulkner *et al.* (2007)	It would leave me feeling tired	Rated 2.1/5 for importance
Fraser *et al*. (2015)	Feel too tired	**74% agreed**
Fraser *et al*. (2015)	Lack of energy	76% agreed
Klingaman *et al.* (2014)	Too tired	**27.8% agreed**
Sylvia *et al.* (2009)	I do not have enough energy	**69% agreed.** Avg. rating = 7.4/10
Ussher (2007)	Too tired	**20% agreed**
(2) Psychological barriers		
*Stress/depression*		
Carpiniello *et al.* (2013)	Unconfident about ability to exercise if sad/stressed	**76.4% agreed**
Klingaman *et al.* (2014)	Stress/depression	**48% agreed**
Ussher (2007)	Unconfident about ability to exercise if sad/stressed	**58% agreed**
*Low motivation*		
Carpiniello *et al.* (2013)	Poor desire	25.4% agreed
Deighton & Addington (2014)	Lack of motivation	‘Sometimes a barrier’
Fraser *et al*. (2015)	Lack of motivation	73% agreed
*Disinterest*		
Bassilios *et al.* (2014)	Disinterest as a barrier	55% of non-vigorous exercisers
Deighton & Addington (2014)	Lack of programmes that interest me	‘Never or sometimes a barrier’
Fraser *et al*. (2015)	Do not enjoy physical activity	**27% agreed**
Klingaman *et al.* (2014)	Do not like exercise	**22.4% agreed**
Sylvia *et al.* (2009)	I do not have enough interest in exercising	**48% agreed.** Avg. rating = 5.5/10
*Self-confidence*		
Deighton & Addington (2014)	Don't like how my body looks	‘Never or sometimes a barrier’
Deighton & Addington (2014)	Failure to achieve exercise goals in the past	‘Never or sometimes a barrier’
Deighton & Addington (2014)	Lack of skills or ability to do a certain type of exercise	‘Never or sometimes a barrier’
Faulkner *et al.* (2007)	I would worry about what other people think of me	Rated 1.4/5 for importance
Faulkner *et al.* (2007)	I would be worried that I would not be very good at it	Rated 2/5 for importance
Fraser *et al*. (2015)	Feel too shy/embarrassed	36% agreed
Fraser *et al*. (2015)	Not the sporty type	29% agreed
Gorczynski *et al.* (2010)	I feel embarrassed if people see me doing it	Rated 1.7/5 on importance scale
Ussher (2007)	Self-consciousness	7% agreed
*Feeling unsafe*		
Carpiniello *et al.* (2013)	Feel unsafe going outdoors	**9% agreed**
Deighton & Addington (2014)	Feeling uncomfortable or intimidated	‘Never or sometimes a barrier’
Deighton & Addington (2014)	Fear of making an existing condition worse	‘Never or sometimes a barrier’
Fraser *et al*. (2015)	Feels unsafe to go outside	**16% agreed**
Klingaman *et al.* (2014)	Safety concerns	**14% agreed**
Ussher (2007)	Feel unsafe going outdoors	**9% agreed**
*Fear of injury*		
Carpiniello *et al.* (2013)	Afraid of getting injured	**5.8% agreed**
Deighton & Addington (2014)	Fear of injury or re-injury	‘Never or sometimes a barrier’
Faulkner *et al.* (2007)	I might injure myself	Rated 2/5 for importance
Fraser *et al*. (2015)	Worried I might get injured	**9% agreed**
Ussher (2007)	Afraid of getting injured	**8% agreed**
(3) Socio-ecological barriers		
*Lack of time*		
Carpiniello *et al.* (2013)	Takes too much time	**23.9% agreed**
Deighton & Addington (2014)	Lack of time	‘Never or sometimes a barrier’
Faulkner *et al.* (2007)	It would take time away from other things	Rated 2.1/5 for importance
Fraser *et al*. (2015)	I do not have enough time	**13% agreed**
Gorczynski *et al.* (2010)	It takes time away from doing other things	Rated 2.7/5 on importance scale
Klingaman *et al.* (2014)	Too little time	**13.9% agreed**
Klingaman *et al.* (2014)	Job/work	5.6% agreed
Klingaman *et al.* (2014)	Daily routine do not include exercise	26.5% agreed
Sylvia *et al.* (2009)	I do not have enough time to exercise	**32% agreed**
Ussher (2007)	Takes too much time	**12% agreed**
*Lack of support*		
Carpiniello *et al.* (2013)	Would receive little help with exercise from others	**65.3% agreed**
Deighton & Addington (2014)	Lack of support from others	‘Never or sometimes a barrier’
Gorczynski *et al.* (2010)	I would need too much help from others	Rated 2.5/5 on importance scale
Klingaman *et al.* (2014)	Lack of support/encouragement	**19.8% agreed**
Ussher (2007)	Would receive little help with exercise from others	**68% agreed**
*Lack of information*		
Carpiniello *et al.* (2013)	Not sure what to do	**15% agreed**
Deighton & Addington (2014)	Lack of knowledge about how to exercise	‘Never or sometimes a barrier’
Faulkner *et al.* (2007)	I don't know how to do physical activities	Rated 1.8/5 for importance
Faulkner *et al.* (2007)	Difficult to find out what to do and where to do it	Rated 2.1/5 for importance
Gorczynski *et al.* (2010)	There is too much I have to learn to do it	Rated 2.3/5 on importance scale
Sylvia *et al.* (2009)	Not know how to exercise/what to do in a gym	**11% agreed.** Avg. rating = 2.9/10
Ussher (2007)	Not sure what to do	**6% agreed**
*Cost*		
Deighton & Addington (2014)	Cost of physical activity programme	‘Sometimes a barrier’
Faulkner *et al.* (2007)	It would cost too much	Rated 2.4/5 for importance
Fraser *et al*. (2015)	Cost	19% agreed
Klingaman *et al.* (2014)	Too little money	24.7% agreed
*Access to facilities*		
Deighton & Addington (2014)	Lack of transportation	‘Never or sometimes a barrier’
Deighton & Addington (2014)	Lack of facilities near by	‘Never or sometimes a barrier’
Fraser *et al*. (2015)	Lack of access to facilities	41% agreed
Klingaman *et al.* (2014)	No place to walk or be active	11.2% agreed
Klingaman *et al.* (2014)	No transport	11.8% agreed
*Training partner*		
Deighton & Addington (2014)	Do not have anyone to go with	‘Never or sometimes a barrier’
Faulkner *et al.* (2007)	I would have to do it by myself	Rated 2.7/5 for importance
Gorczynski *et al.* (2010)	I would have to do it by myself	Rated 2.8/5 on importance scale
Sylvia *et al.* (2009)	I have no one to exercise with	Rated 2.6/10 for relevance

a **Bold** indicates inclusion in meta-analysis.

### Physical health motivations

**Fig. 2.**
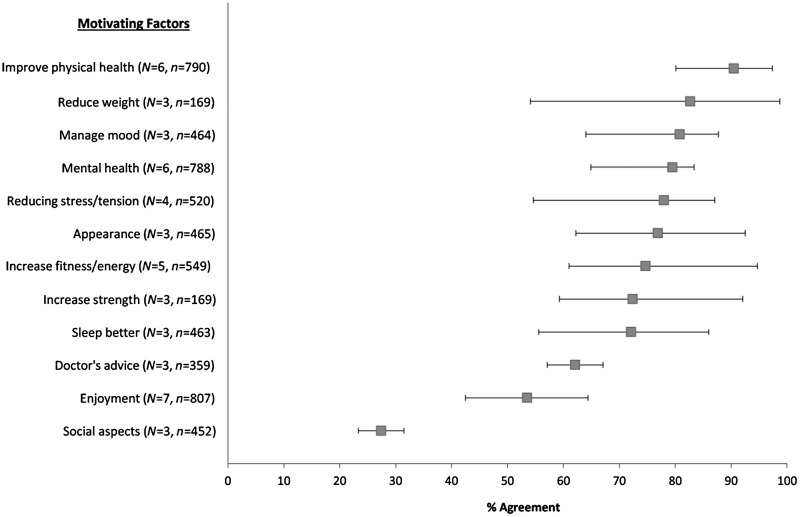
Proportional meta-analyses of motivating factors for exercise in severe mental illness. The forest plot shows the % of patients agreeing with each motivating factors (box points) and the 95% confidence intervals (horizontal lines). Individual study items used in meta-analyses are shown in Table 1.

Meta-analyses of proportional data are displayed in Fig. 2. The most endorsed reason for exercising was to improve general physical health; endorsed by 91% of people with SMI (*N* = 6, *n* = 790, 95% CI 80–94, *Q* = 81, *p* < 0.01, *I*^2^ = 94%). Two studies which examined motivations for exercise using Likert scales also found that general health improvement ranked higher than all other options (Faulkner *et al.* 2007; Gorczynski *et al.* 2010).

Increasing fitness/energy was the most widely assessed physical health motivation (*N* = 5, *n* = 549). This was a motivating factor for 75% of respondents (95% CI 64.9–83.4, *Q* = 19, *p* < 0.01, *I*^2^ = 79%) and ranked as ‘highly important’ in three Likert-scale studies (Faulkner *et al.* 2007; Sylvia *et al.* 2009; Gorczynski *et al.* 2010). ‘Improving appearance’ and ‘losing weight’ were examined in only three studies each, but received high rates of endorsement of 77% (*n* = 465, 95% CI 64–88, *Q* = 13.3, *p* < 0.01, *I*^2^ = 85%) and 83%, respectively (*n* = 169, 95% CI 54–99, *Q* = 30, *p* < 0.01, *I*^2^ = 93%). ‘Improving strength’ averaged 72% endorsement (*N* = 3, *n* = 169, 95% CI 55–87, *Q* = 10, *p* < 0.01, *I*^2^ = 81%).

### Psychological motivations

As shown in Fig. 2, overall mental health, reducing stress and managing mood were equally popular motivating factors, with 80% (*N* = 6, *n* = 788, 95% CI 62–93, *Q* = 134, *p* < 0.01, *I*^2^ = 96%), 78% (*N* = 4, *n* = 520, 95% CI 59–92, *Q* = 50, *p* < 0.01, *I*^2^ = 94%) and 81% (*N* = 3, *n* = 464, 95% CI 62–93, *Q* = 32, *p* < 0.01, *I*^2^ = 94%) of patients agreeing, respectively. Improved sleeping patterns was a motivating factor for 72% of patients (*N* = 3, *n* = 464, 95% CI 55.6–86, *Q* = 20, *p* < 0.01, *I*^2^ = 90%). Enjoyment of exercise was only endorsed by 54% of respondents (*n* = 807, 95% CI 42.5–64.6, *Q* = 53, *p* < 0.01, *I*^2^ = 89%). Likert scales studies also found that mental health benefits and enjoyment of exercise scored moderate-to-high for importance as reasons for exercise. The benefits of exercise for self-confidence were assessed in five studies. Although unsuitable for meta-analysis, five studies which assessed the benefits of exercise for self-confidence showed that this is a broadly accepted and valued reason to exercise (See Table 1).

### Socio-ecological motivations

Social aspects of exercise seen as motivating factors by 27% of patients (*N* = 3, *n* = 452, 95% CI 23–32, *Q* = 0.1, *p* < 0.097, *I*^2^ = 0%). In Likert-scale studies, social aspects scored the lowest of all options presented (Sylvia *et al.* 2009; Gorczynski *et al.* 2010; Kane *et al.* 2012). Similarly, only a minority of participants saw ‘improving daily routine’ as an important reason for exercise (Sylvia *et al.* 2009; Wynaden *et al.* 2012). In contrast, three independent studies found that ‘professional support’ was perceived as a motivating factor for increasing exercise by the majority of patients (Ussher, 2007; Sylvia *et al.* 2009; Carpiniello *et al.* 2013).

### Physical health barriers

**Fig. 3.**
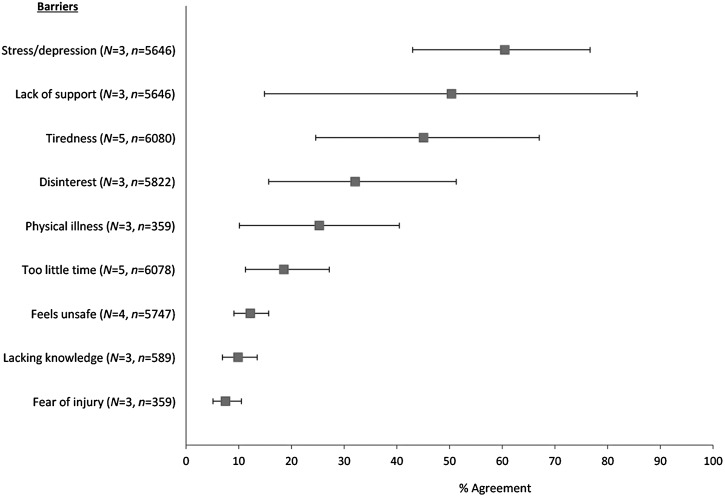
Proportional meta-analyses of barriers to exercise in severe mental illness. The forest plot shows the % of patients experiencing each barrier (box points) and the 95% confidence intervals (horizontal lines). Individual items combined for meta-analysis are shown in Table 2.

Fig. 3 shows meta-analyses of barriers towards exercise. Physical illness and poor health was a barrier for 25% of participants (*N* = 3, *n* = 359, 95% CI 10–41, *Q* = 64, *p* < 0.01, *I*^2^ = 92%). Tiredness/low energy was more common, reported by 45% of patients (*N* = 5, *n* = 6080, 95% CI 25–67, *Q* = 322, *p* < 0.01, *I*^2^ = 99%) and rated as 7.4/10 on relevance scales (Sylvia *et al.* 2009). Two studies also showed that patients with long-term schizophrenia were more affected by tiredness than healthy controls (Carpiniello *et al.* 2013; Klingaman *et al.* 2014). However, this difference did not exist between patients with first-episode psychosis and healthy controls (Deighton & Addington, 2014).

### Psychological barriers

Proportional meta-analyses showed substantial differences in psychological barriers. ‘Stress/depression’ was a barrier to exercise for 61% of respondents (*N* = 3, *n* = 5646, 95% CI 43–77, *Q* = 48, *p* < 0.01, *I*^2^ = 96%), whereas ‘disinterest in exercise’ was a barrier for only 32% (*N* = 3, *n* = 5822, 95% CI 16–51, *Q* = 96, *p* < 0.01, *I*^2^ = 98%). Feeling unsafe and fears of injury were even less common, at 12% (*N* = 4, *n* = 5747, 95% CI 9–16, *Q* = 7, *p* = 0.07, *I*^2^ = 57%) and 8% (*N* = 3, *n* = 359, 95% CI 5–11, *Q* = 0.9, *p* = 0.64, *I*^2^ = 0%), respectively. Data from five studies assessing confidence-related barriers was unsuitable for meta-analyses, but collectively showed that this was only a concern for a minority of participants (7–36%), and to a limited extent; consistently scoring <2/5 on Likert scales of importance (Table 2).

Data on ‘low motivation’ was also unsuitable for proportional meta-analysis. However, all three studies which assessed this found that motivational deficits were among the most common psychological barriers towards exercise (Carpiniello *et al.* 2013; Deighton & Addington, 2014; Fraser *et al.* 2015). Furthermore, patients with long-term schizophrenia experienced motivational barriers significantly more than healthy controls (Carpiniello *et al.* 2013). Again, however, there was no significant difference in the early stages of illness (Deighton & Addington, 2014).

### Socio-ecological barriers

The most frequently experienced practical barrier was a ‘lack of support’, reported by 50% of respondents (*N* = 3, *n* = 5646, 95% CI 15–86, *Q* = 240, *p* < 0.01, *I*^2^ = 99%). This was significantly more prevalent among schizophrenia patients than healthy controls (Carpiniello *et al.* 2013; Klingaman *et al.* 2014). People with first-episode psychosis also scored these items higher than controls, although differences were not statistically significant (Deighton & Addington, 2014). ‘Lack of training partner’ was a moderately ranked barrier, but was regarded as significantly more important by those patients who were interested in increasing their exercise (Faulkner *et al.* 2007).

‘Lack of time’ was the most widely investigated practical barrier, although only 19% of respondents identified this as a barrier (*N* = 5, *n* = 6078, 95% CI 11.3–27.2, *Q* = 68, *p* < 0.01, *I*^2^ = 94%). Three studies using Likert scales also found that time-related barriers were mostly unimportant (Faulkner *et al.* 2007; Gorczynski *et al.* 2010; Deighton & Addington, 2014). Furthermore, ‘lack of time’ was significantly less of a barrier for people with SMI than for healthy controls (Deighton & Addington, 2014; Klingaman *et al.* 2014). Only 10% of patients felt that ‘lack of exercise information’ was a barrier (*n* = 589, 95% CI 7–14, *Q* = 3.4, *p* = 0.18, *I*^2^ = 42%). Additional data (unsuitable for meta-analysis) on cost and accessibility of exercise services indicated these were of low importance (See Table 2).

## Discussion

The purpose of this study was to examine the motivating factors and barriers towards exercise among people with SMI, in order to inform the design and delivery of interventions aiming to increase exercise participation. A total of 12 studies (of 6431 psychiatric patients with predominantly schizophrenia/schizoaffective disorders) were identified. As nine of the 12 studies reviewed had been conducted from 2013 onwards, the evidence/data presented can be considered timely and up-to-date.

Our results show that the primary incentive for engaging in exercise was to improve physical health (Fig. 2). Specifically, weight loss was the single most popular reason for participating in exercise, comparable to the motivating factors identified by the general population (Sherwood & Jeffery, 2000), and unsurprising given the high rates of overweight and obesity among people with SMI (Vancampfort *et al.* 2015*b*). Although weight management can be a key motivating factor for initiating an exercise programme, it is important to note (*a*) the relatively modest contribution of physical activity to weight loss beyond that achieved through dietary interventions (Haskell *et al.* 2007), and (*b*) that improvements in mental and physical health outcomes in response to exercise interventions are often achieved independent of weight loss (Firth *et al.* 2015). While weight management may be an important motivating factor for people with SMI to commence an exercise programme, education and support should be provided to ensure long-term adoption and maintenance regardless of any change in body weight achieved. Furthermore, if weight loss is a primary aim, dietary interventions must be provided as part of best-practice lifestyle interventions (Ward *et al.* 2015).

The high endorsement of ‘fitness’ as an incentive is encouraging, since this is readily improved by exercise interventions in SMI (Vancampfort *et al.* 2015*a*, 2016*b*), and is more predictive of cardiovascular disease than any other aspect of metabolic health (Myers *et al.* 2004; Hu *et al.* 2005). Health promotion programmes should therefore emphasize the benefit of fitness in order to maximize uptake of exercise in this patient group. Furthermore, interventions should ideally be designed by exercise professionals to ensure that they meet basic principles of exercise prescription, in order to exert significant physiological effects and enable patients to achieve realistic fitness goals.

Patients also valued the psychological effects of exercise, and 75% of patients viewed stress reduction/mood enhancement as motivating factors. Recent meta-analyses have shown that exercise can significantly improve psychological well-being among people with SMI and reduce depression (Rosenbaum *et al.* 2014; Firth *et al.* 2015). However, the present study also found that stress, depression and low energy often also act as barriers towards exercise.

The most prominent socio-ecological barrier identified across the studies included in this review was a ‘lack of support’. Nonetheless, the majority of patients felt that exercise supervision would enable them to exercise more (Ussher, 2007; Sylvia *et al.* 2009; Carpiniello *et al.* 2013). This is congruent with the qualitative literature, within which patients with SMI have stipulated that adequate support can overcome many of the barriers faced towards exercise (Soundy *et al.* 2014*b*; Firth *et al.* 2016*b*).

Although unsupervised interventions which use less resource-intensive methods (such as education or behavioural change techniques) may seem more cost effective than supervised exercise, this may not be the case for people with SMI. Several recent meta-analyses of exercise interventions in this population have shown that interventions which provide professional support have better adherence to physical activity and significantly greater effects on cardiorespiratory fitness (Vancampfort *et al.* 2015*c*, 2016*b*; Stubbs *et al.* 2016*c*). Since both physical activity and fitness are strong predictors of cardiovascular risk and all-cause mortality (Hu *et al.* 2005; Kodama *et al.* 2009), supervised interventions which effectively target these variables may ultimately prove more financially worthwhile for improving long-term health outcomes (Vancampfort *et al.* 2015*c*, 2016*b*).

Previous intervention studies have further shown that whereas exercise access and advice is ineffective for increasing physical activity in SMI (Archie *et al.* 2003; Bartels *et al.* 2013), providing adequate social support does enable patients to achieve sufficient levels of moderate-to-vigorous exercise (Bartels *et al.* 2013; Firth *et al.* 2016*c*). Although there is currently a lack of cost-effectiveness research examining supervised exercise in SMI, financial reports of exercise interventions for diabetes, mild depression and heart disease indicate that professionally delivered training programmes produce large economic benefits from avoided health system costs (Deloitte Access Economics, 2015).

### Limitations

A strength of these findings is the large number of patients (*n* = 6431) included in the review. Within this, there was also substantial ethnic diversity within the included samples, with 50% belonging to minority groups. However, all of the studies were conducted in western, developed countries, and thus no studies have examined barriers towards exercise among people with SMI in Asia or developing countries. Furthermore, no studies examined differences in motivations or barriers towards exercise between the different ethnic groups within their respective samples. This gap in the literature should be given further consideration in future research, as studies in the general population have shown that beliefs about exercise, and primary reasons for engaging in physical activity, differ significantly between ethnic groups even within the same country (Dergance *et al.* 2003; Shiu-Thornton *et al.* 2004). Specifically, those in minority ethnic groups may face additional challenges towards exercise, such as feeling unsafe in their neighbours (Fahlman *et al.* 2006) or lacking opportunity to engage in culturally appropriate physical activity (Caperchione *et al.* 2009). Thus, efforts should be undertaken to identify and provide acceptable physical activity interventions for ethnically diverse populations.

Despite the large total sample, one limitation of this review is that some of the motivations and barriers assessed in meta-analyses were examined by as few as three studies. Additionally, some eligible studies did not provide any proportional data, and thus were not included in the meta-analysis at all. Nonetheless, a full systematic review of each eligible study was also undertaken, for consideration alongside the meta-analytic outputs, in order to provide a complete account of all relevant findings.

It should also be considered that the large majority of patients (85%) in this meta-analysis had a diagnosis of schizophrenia, while bipolar disorder and major depressive disorder were relatively under-represented among the eligible studies. Thus, future research should examine if the same motivations and barriers towards exercise identified in this review also generalize to patients with SMIs other than schizophrenia. An online survey study of individuals with high depressive symptoms (but without a confirmed SMI) indicates that our findings will generalize beyond schizophrenia, as the most common barriers towards exercise reported by these individual were again low mood and fatigue (Busch *et al.* 2015), as was observed in our SMI samples (Fig. 3).

A final limitation is that results are based on self-reported data, derived from questionnaires and surveys administered to patients. Therefore, the results could be affected by response bias, or participants lacking sufficient interest/experience with exercise to accurately describe the barriers faced. The findings from patients’ self-report in this study are also congruent with health professionals’ opinions, who also acknowledge the importance of social support in overcoming various barriers towards regular exercise (Soundy *et al.* 2014*c*).

## Conclusion

People with SMI value exercise for its ability to improve physical health and appearance, and the psychological benefits. However, mental health symptoms, tiredness and insufficient support present substantial barriers for the majority of patients. Taking this into account, exercise training programmes for people with SMI should be designed to improve exercise capacities and cardiorespiratory fitness, while also providing the necessary levels of supervision or assistance for each patient to overcome psychological barriers and achieve their goals. Such interventions would be motivating and rewarding for patients, resulting in higher levels of exercise engagement. This, in turn, could improve physical health outcomes and facilitate functional recovery in SMI.

## Supplementary material

For supplementary material accompanying this paper visit http://dx.doi.org/10.1017/S0033291716001732. click here to view supplementary material
