# Supplementary material for: Motivating factors and barriers towards exercise in severe mental illness: a systematic review and meta-analysis
Source: Psychol Med. 2016 Aug 9;46(14):2869–81. doi: 10.1017/S0033291716001732 (PMC5080671; doi:10.1017/S0033291716001732)
Supplement: Supplementary file 1 [file S0033291716001732sup001.zip › Supplementary Table 2. Included studies.doc]

| **Supplementary Table S2.** *Characteristics of Included Studies* | | | | | | |
| --- | --- | --- | --- | --- | --- | --- |
| **Reference** | **N** | **Country** | **Setting** | **Age (years)** | **Diagnostic info** | **Data for meta-analyses** |
| **Bassilios et al. 2014** | 49 | Australia | Community mental health | 39 | SZ / SZ-AF | Data only suitable for systematic review |
| **Carpiniello et al. 2013** | 138 | Italy | Community mental health | 50 | 29% SZ, 28% MDD, 25% BP | Motivations and barriers - reported in manuscript |
| **Deighton et al. 2014** | 40 | Canada | Early Intervention Services | 19.8 | ‘First-episode psychosis' | Motivating factors - proportional data obtained from author |
| **Faulkner et al. 2007** | 109 | Canada | Mental health and addiction facility | 46.5 | 96% SZ, MDD or BP | Data only suitable for systematic review |
| **Firth et al. 2016** | 28 | UK | Early Intervention services | 25.5 | ‘First-episode psychosis' | Motivating factors - reported in manuscript |
| **Fraser et al. 2015** | 101 | Australia | Inpatient unit | 40.7 | 10% SZ, 61% MDD, 18% BP | Motivations and barriers - reported in manuscript |
| **Gorczynski et al. 2010** | 54 | Canada | Outpatient satellite clinic | 46.7 | “serious mental illness” | Data only suitable for systematic review |
| **Kane et al. 2012** | 12 | USA | Outpatient clinics | 43.25 | SZ | Data only suitable for systematic review |
| **Klingaman et al. 2014** | 5388 | USA | ‘Veteran Affairs’ database | 55 | SZ | Barriers - reported in manuscript |
| **Sylvia et al. 2009** | 86 | USA | Partial hospital program | 37 | SZ, MDD or BP | Motivations and barriers - obtained from author (for 336 participants) |
| **Ussher et al. 2007** | 120 | UK | Inpatients and outpatients | 42.6 | “severe mental illness” | Motivations and barriers - reported in manuscript |
| **Wynaden et al. 2012** | 56 | Australia | Forensic mental health services | 19-50 | “mostly psychotic illness” | Motivating factors - reported in manuscript |
